# Recovery of adrenal function in a patient with confirmed Addison's disease

**DOI:** 10.1530/EDM-13-0070

**Published:** 2013-12-01

**Authors:** M Baxter, S Gorick, F M Swords

**Affiliations:** 1 Norfolk and Norwich University Hospital NHS Foundation TrustNorwichUK

## Abstract

Addison's disease is a condition characterised by immune-mediated destruction of the adrenal glands leading to a requirement of lifelong replacement therapy with mineralocorticoid and glucocorticoid. We present a case of a 53-year-old man who presented at the age of 37 years with nausea, fatigue and dizziness. He was found to have postural hypotension and buccal pigmentation. His presenting cortisol level was 43 nmol/l with no response to Synacthen testing. He made an excellent response to conventional replacement therapy with hydrocortisone and fludrocortisone and then remained well for 16 years. On registering with a new endocrinologist, his hydrocortisone dose was revised downwards and pre- and post-dose serum cortisol levels were assessed. His pre-dose cortisol was surprisingly elevated, and so his dose was further reduced. Subsequent Synacthen testing was normal and has remained so for further 12 months. He is now asymptomatic without glucocorticoid therapy, although he continues on fludrocortisone 50 μg daily. His adrenal antibodies are positive, although his ACTH and renin levels remain elevated after treatment. Addison's disease is generally deemed to lead to irreversible cell-mediated immune destruction of the adrenal glands. For this reason, patients receive detailed counselling and education on the need for lifelong replacement therapy. To our knowledge, this is the third reported case of spontaneous recovery of the adrenal axis in Addison's disease. Recovery may therefore be more common than previously appreciated, which may have major implications for the treatment and monitoring of this condition, and for the education given to patients at diagnosis.

**Learning points:**

- Partial recovery from Addison's disease is possible although uncommon.
- Patients with long-term endocrine conditions on replacement therapy still benefit from regular clinical and biochemical assessment, to revisit optimal management.
- As further reports of adrenal axis recovery emerge, this may influence the counselling given to patients with Addison's disease in the future.

## Background

Addison's disease is characterised by cell-mediated immune destruction of the adrenal glands, either in isolation or as part of a polyglandular autoimmune syndrome. This process is widely deemed to be irreversible. As such, patients with Addison's disease require extensive education on the importance of lifelong replacement therapy with glucocorticoid and mineralocorticoid. We present a new case of autoimmune Addison's disease where adrenal function appears to be restored over time. This appears to be the second ever recorded case of spontaneous recovery. It is uncertain whether this is truly a very rare phenomenon, or whether this recovery might be more common with modern dosing regimens if patients with Addison's are regularly reassessed.

## Case presentation

A 37-year-old male soldier presented in 1997 with classical Addison's disease. He presented with fatigue, nausea, vomiting, weight loss, malaise and dizziness. On examination, marked skin and buccal hyperpigmentation and orthostatic hypotension were diagnosed. There was no relevant family history. Baseline biochemistry was highly suggestive of Addison's disease: sodium 132, potassium 6.1, urea 8.5 mmol/l and random cortisol 43 nmol/l. There was no response to 250 mg Synacthen test: cortisol level rose to 56 nmol/l at 30 min. Adrenal antibodies and ACTH and renin levels were not checked at that time.

He was treated conventionally with hydrocortisone 20 mg on waking, 10 mg midafternoon and fludrocortisone 50 μg daily, resulting in an excellent recovery.

**Figure 1:**
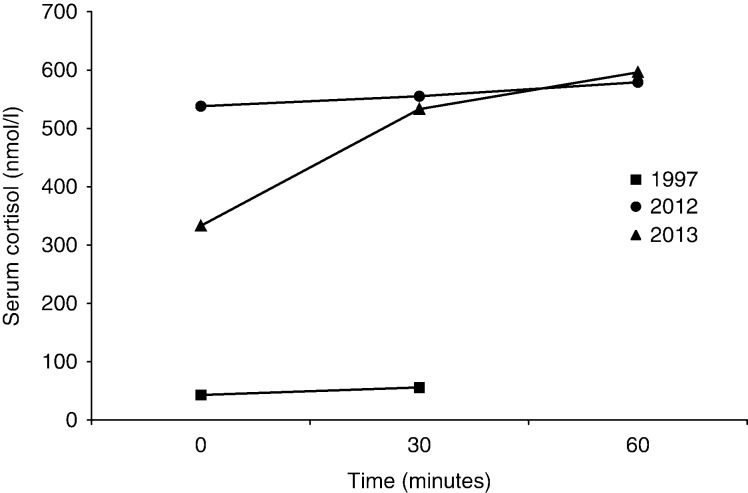
The *x* axis shows the serum cortisol response in nmol/l to standard short Synacthen testing. The results (0 and 30 min only) at diagnosis in 1997 are shown in the filled squares, the results in 2012 during treatment but 24 h following any hydrocortisone dose are shown in the filled circles, and his latest results in 2013 are shown in the filled triangles.

He then remained well for the next 12 years, but on registering with a new endocrinologist, his hydrocortisone requirement was reassessed. Gradual dose reduction was suggested, and pre- and post-dose serum cortisol levels were also checked. His pre-dose cortisol was surprisingly elevated at 359 nmol/l, rising to 1181 nmol/l 2 h post-hydrocortisone dose. His hydrocortisone dose was therefore reduced to 20 mg on waking and 5 mg midafternoon due to patient's reluctance to reduce his morning dose further. Repeat Synacthen testing was then performed and confirmed to be normal (Fig. 1). Hydrocortisone dose was therefore cautiously and slowly reduced further. He noticed no change in his symptoms and continued to walk 6 miles to work daily, with an ongoing normal blood pressure and serum electrolytes on 5 mg hydrocortisone on waking only. On repetition of Synacthen testing, a rise in cortisol level from 335 to 596 nmol/l was observed. Hydrocortisone dose was therefore completely withdrawn and the patient remains well after 4 months of treatment. After treatment, his 0900 h ACTH level was initially elevated at 86 ng/l but has now normalised at 13 ng/l. Renin remains elevated 24 h post-fludrocortisone dose, and so this has been continued. Adrenal antibody tests are positive.

## Investigation

The 1997 initial Synacthen test results: peak cortisol 56 nmol/l at 30 min. 03/2012 Pre-dose cortisol 359 nmol/l, rising to 1181 nmol/l 2 h post-hydrocortisone dose (=15 years after starting treatment for confirmed Addison's disease). 09/2012 Synacthen test results, taken 24 h after last dose of 20 mg hydrocortisone: baseline cortisol 538 nmol/l, 30-min cortisol 555 nmol/l and 60-min cortisol 579 nmol/l. 09/2012 ACTH 86 ng/l, renin 158 mU/l 24 h after any glucocorticoid or mineralocorticoid doses. 05/2013 Synacthen test: baseline cortisol 335 nmol/l, 30-min cortisol 533 nmol/l and 60-min cortisol 596 nmol/l. ACTH 13 ng/l and renin 203 mU/l.

Autoimmune screening was not performed at diagnosis. In 2013 thyroid peroxidase and tissue transglutaminase antibodies are negative. Anti-smooth muscle antibodies are also positive at a dilution of 1/320 but the rest of his autoantibody screening including anti-nuclear antibodies are negative. Adrenal antibodies are positive as tested using a semi-automated immunofluorescence method (using pre-coated monkey adrenal slides and a commercially available conjugate, both from INOVA Diagnostics San Diego, California, US). Unfortunately, they had never been tested previously.

## Treatment

Hydrocortisone was cautiously withdrawn completely in June 2013 following several months on very low-dose therapy with no adverse effects and a persistently normal Synacthen test. The patient also remains on fludrocortisone 50 μg on waking in view of the persistently elevated renin when this has been omitted for testing.

## Outcome and follow-up

Hydrocortisone was stopped completely in June 2013, 16 years after the initial diagnosis of Addison's disease was made. The patient remains asymptomatic and well. The patient also has an emergency supply of hydrocortisone for both oral and i.m. administration in the event of intercurrent illness. He and his family have also received extensive education from a specialist endocrine nurse to ensure that they understand the unusual nature of his situation. He will be followed closely for the development of other autoimmune endocrine conditions, and with regular monitoring of his early morning cortisol levels with repeat Synacthen testing when indicated to detect any deterioration in his adrenal function in future.

We also plan to check his aldosterone level and to consider stopping his fludrocortisone dose if this is detectable, and the patient remains well, although unfortunately, this has not been previously assessed.

## Discussion

This represents the third and most clear-cut documented case of a patient with confirmed Addison's disease with spontaneous recovery. Smans *et al*. (1) documented a patient who was able to stop treatment after 7 years, but with ongoing subnormal response to Synacthen testing. More recently, a second case was described with biochemical Addison's disease (peak cortisol 175 nmol/l) presenting in pregnancy and complicated by concurrent use of inhaled steroids and enzyme-inducing drugs (2). This patient had fluctuating responses to Synacthen on follow-up and has required intermittent glucocorticoid replacement therapy. We describe the first case with unequivocally abnormal biochemistry at diagnosis (peak cortisol 56 nmol/l) and unequivocably normal biochemistry after 16 years of treatment (peak cortisol 579 nmol/l).

This raises several interesting issues. The first is that endocrinologists need to be more vigilant at continually reassessing patients with established or presumed diagnoses, particularly if these have been made elsewhere. In this case, a patient with a very well-defined and confirmed biochemical diagnosis of Addison's was reassessed due to his somewhat old-fashioned and supraphysiological glucocorticoid replacement regimen. This led to more detailed biochemical assessment than had previously been undertaken and revealed that he no longer required daily glucocorticoid replacement therapy at all. It is now impossible to determine how many years of potentially unnecessary glucocorticoid therapy this patient had been exposed to if any. It is reassuring that there were no clinical signs of supraphysiological glucocorticoid treatment in this case, although obvious side effects may have led the diagnosis to be challenged earlier.

A recent study performed short Synacthen tests on 27 patients with established Addison's disease. The authors did not uncover any other examples of spontaneous recovery (3). It is possible that this cohort was very small and that the phenomenon is still more common than previously thought. It is also unknown whether any specific characteristics might predict which patients could recover over time. The patient described here continues to have positive adrenal antibodies, which might be taken as a negative predictor of recovery. On the other hand, he has no family history, no other autoimmune conditions and ongoing normal thyroid function that might be a more positive sign.

Furthermore, it is possible, that a subset of patients with Addison's disease and the potential for adrenal recovery might only exhibit this recovery when their glucocorticoid dose is reduced into more physiological ranges. Recovery was only detected after the dose of glucocorticoid was slowly reduced in the first reported case (1). In another case, subclinical hypoadrenalism in association with Graves' disease was reported to improve after treatment of the patient with high-dose steroids for their ophthalmopathy (4). In this case, the patient had been taking 30–40 mg hydrocortisone for many years but was not exposed to any other or higher dose immunosuppressives that might have ‘treated’ an immune adrenalitis and so contributed to his subsequent recovery. It is possible that the reduction in his total daily dose allowed his recovery, by releasing his hypothalamo-pituitary–adrenal axis from supraphysiological suppression, but the dose used, length of time since diagnosis, the early increase in ACTH on treatment withdrawal and the continued presence of positive adrenal antibodies go against this mechanism.

Other studies have explored patients with positive adrenal antibodies but without overt Addison's disease (5). Anti-adrenal antibodies are associated with Addison's disease, but in this study of 20 patients with positive antibodies, some showed spontaneous disappearance of the antibodies, and no subsequent Addison's, some showed a progression through subclinical hypoadrenalism and then overt Addison's disease with persistently positive antibodies, while others developed clear Addison's disease but with subsequent loss of their antibodies. No episodes of recovery were detected. This seems to offer the most plausible mechanism to explain these cases: perhaps analogous to thyroid disease, adrenal autoantibodies also fluctuate in character and quantity leading to unpredictable adrenal function over time.

Finally, it is possible that his initial confirmed glucocorticoid deficiency was non-immune in nature. The pigmentation at presentation, suggesting elevated ACTH, goes against the possibility of adrenal suppression from exogenous steroid exposure. Similarly, malignancy, tuberculosis, adrenal haemorrhage or infarction would be expected to be clinically obvious either at presentation or over such a long follow-up period, and the presence of adrenal and other autoantibodies now does support the supposition that this was immune-mediated Addison's disease. However, this cannot be confirmed 100%.

In summary, recovery from Addison's disease is possible, although we do not yet know whether this will be sustained long term in this case. Similarly, we have no current models to predict which patients are more likely to recover than others. Vigilance in the follow-up of patients with long-term endocrine conditions is therefore paramount. Secondly, all opportunities to revise glucocorticoid dosing downwards where possible should be sought. Finally, this case demonstrates the importance of patient involvement in the management of long-term conditions and has absolutely relied on detailed educational input from a specialist nurse to maintain his safety.

## Patient consent

Consent obtained.

## Author contribution statement

Dr M Baxter is the Endocrinology Foundation Year 1 Doctor and prepared initial drafts of the manuscript. S Gorick is the specialist endocrine nurse who performed all patient education and Synacthen tests and aided in the manuscript preparation. Dr F Swords is the consultant endocrinologist and named physician who was responsible for the patient.
